# Total Protein Extraction for Metaproteomics Analysis of Methane Producing Biofilm: The Effects of Detergents

**DOI:** 10.3390/ijms150610169

**Published:** 2014-06-06

**Authors:** Hung-Jen Huang, Wei-Yu Chen, Jer-Horng Wu

**Affiliations:** Department of Environmental Engineering, National Cheng Kung University, No. 1, University Road, Tainan City 701, Taiwan; E-Mails: hjhuang1976@yahoo.com.tw (H.-J.H.); weiuie.chen@gmail.com (W.-Y.C.)

**Keywords:** metaproteomic, biofilm, protein recovery

## Abstract

Protein recovery is crucial for shotgun metaproteomics to study the *in situ* functionality of microbial populations from complex biofilms but still poorly addressed by far. To fill this knowledge gap, we systematically evaluated the sample preparation with extraction buffers comprising four detergents for the metaproteomics analysis of a terephthalate-degrading methanogenic biofilm using an on-line two-dimensional liquid chromatography tandem mass spectrometry (2D-LC-MS/MS) system. Totally, 1018 non-repeated proteins were identified with the four treatments. On the whole, each treatment could recover the biofilm proteins with specific distributions of molecular weight, hydrophobicity, and isoelectric point. The extraction buffers containing zwitterionic and anionic detergents were found to harvest the proteins with better efficiency and quality, allowing identification up to 76.2% of total identified proteins with the LC-MS/MS analysis. According to the annotation with a relevant metagenomic database, we further observed different taxonomic profiles of bacterial and archaeal members and discriminable patterns of the functional expression among the extraction buffers used. Overall, the finding of the present study provides first insight to the effect of the detergents on the characteristics of extractable proteins from biofilm and the developed protocol combined with nano 2D-LC/MS/MS analysis can improve the metaproteomics studies on microbial functionality of biofilms in the wastewater treatment systems.

## 1. Introduction

Biofilms are the structured consortia of trophic groups of microorganisms grown in a close approximation on the surface of supporting media, which can be involved an important biological process for water/wastewater purification, bioenergy recovery, and environmental remediation. In biofilms, the cellular activities can be regulated through signaling based gene expression and nutrient exchange associated with cell-to-cell and cell-to-environment interactions [1]. Therefore, understanding of *in situ* functionality of microbial populations provides fundamental information for improving the performance of biofilm-related processes. 

Metaproteomics is a suitable approach for a global protein identification of biofilm functionality from various environments, as it allows the study of metabolic activities of single species or complex environmental communities in different environmental matrices. The approach applied to study the complex environmental samples is still in the early stage of development, and highly relies on the analytical throughput and availability of (meta)genomic database [2,3]. Similar to any other proteomic experiments, a metaproteomic analysis undergoes protein recovery from the studied materials, enzymatic digestion, mass-based protein identification, and annotation to generate a dataset that can provide intensive insight to microbial community composition, functionality and activity. Prior to analysis with tandem mass spectrometry (MS/MS), the total proteins recovered from a biofilm sample are firstly separated via one- or two-dimensional gel electrophoresis, and enzymatically in-gel fragmented [4]. The other strategy where the protein extract is in solution digested and then separated through liquid chromatography (LC) provides a large-scale capability of protein identification [5]. The off-gel proteomics has been successfully applied to pure and mixed culture systems [6,7,8,9]. However, the proteome from a biofilm sample is inherently complicated, because the proteins of different abundance are produced by numerous microorganisms with highly complex matrix of *extracellular polymeric substances*. In this regard, the sample preparation such as protein extraction and purification to achieve efficiency for the environmental samples is very challenging [7,10]. The protein extraction efficiency depends both on the much on the matrix reactivity and used extractants [4,5,10,11,12]. Recently, several studies have addressed the efficacy of several extraction protocols specific to the biofilm samples for a downstream analysis with two-dimensional electrophoresis-MS/MS [5,13], which with LC-MS/MS [7,14], showing the importance of the protein extraction to achieve an efficient, unbiased metaproteomic analysis. Among the extraction procedures, the detergents are indispensable solubilizing agents and can help the extraction efficiency of the proteins from microbial cells [15,16]. The effects of the detergents on harvesting the proteins from a pure culture had been reported [16,17]. However, little is known about their efficiency in protein extraction from biofilm.

Methane is one of renewable energy sources and can be largely produced from the anaerobic bioreactors with various microbial groups in the decomposition of organic substances. For example, terephthalic acid, an important raw material used in plastic, textile and petroleum-based industries, can be degraded by fermentative syntrophic bacteria to acetate and H_2_/CO_2_, which are immediately used by acetotrophic and hydrogenotrophic methanogens to generate methane [4,18]. To observe the global expression of microbial functions within the terephthalate-degrading biofilm, in a previous study, biofilm proteome was obtained and analyzed using one-dimensional gel-based proteomics [4]. The results revealed a distinctive distribution pattern of *in situ* microbial functions and confirmed the key proteins involved in the metabolic pathways of terephthalate degradation to methane formation. Because the number of identified proteins is still low and loss of proteins was observed with the used protein extraction protocol during experiments, protein extraction with better recovery efficiency in conjugation with on-line two-dimensional LC-MS/MS should be developed in order to detect more proteins, providing deeper insight on the functionality of biofilm proteome. In this study, we systematically evaluated the effects of detergents of different types, such as anionic, neutral, zwitterionic and mixed, on recovering the proteins from biofilm of an anaerobic bioreactor. 

## 2. Results and Discussion

**Figure 1:**
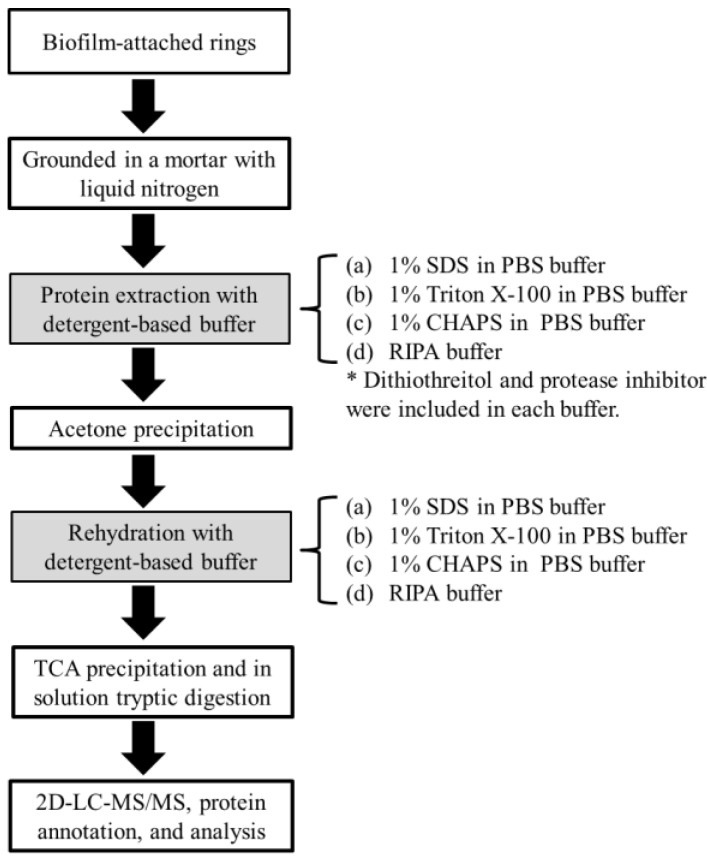
Schematic workflow for protein extraction from biofilms with detergent-containing buffers. SDS (sodium dodecyl sulfate), PBS (phosphate buffered saline), CHAPS (3-[(3-cholamidopropyl)dimethylammonio]-1-propanesulfonate), RIPA (radioimmunoprecipitation assay buffer), TCA (trichloroacetic acid) and 2D-LC-MS/MS (two-dimensional liquid chromatography tandem mass spectrometry).

In this study, we used four different buffers in the trichloroacetic acid (TCA)-based extraction protocol that is commonly used for studies of shotgun metaproteomics [11] to recover total proteins from the methane-producing biofilm grown on the ceramic rings from an anaerobic fixed-film reactor fed with terephthalate as the sole substrate at 50 °C [4]. The biofilm was cultivated for a long time and contained various *Bacteria* and *Archaea* methanogen species producing methane from the degradation of terephthalate [4]. The buffers included an anionic detergent, sodium dodecyl sulfate (SDS, 1%), a neutral detergent, triton X-100 (Triton, 1%), a zwitterionic detergent, 3-[(3-cholamidopropyl)dimethylammonio]-1-propanesulfonate (CHAPS, 1%), and a mixture of anionic and neutral detergents (Nonidet P-40, 0.1% and Sodium deoxycholate, 0.5%, and SDS, 0.1%), RIPA. The obtained proteins were tryptically digested and the produced peptide fragments were analyzed using on-line two-dimensional HPLC-ESI-MS/MS (Figure 1). 

### 2.1. Yield and Number of Proteins Recovered from Methanogenic Biofilm

Concentrations of proteins extracted from biofilm material with the four treatments ranged from 884.8 ± 221.0 to 1182.9 ± 196.6 μg·g^−1^, and the SDS buffer recovered a higher amout of proteins than other three buffers (Table 1), despite being not significant (*t*-test, *p* = 0.23). This result is consistent with previous studies, showing that the anionic SDS extracted more amounts of total protein from bacterial cultures and soil samples than other ionic detergents [15,17]. In total, 591, 473, 418 and 323 proteins were identified using the SDS, RIPA, CHAPS, and Triton buffers, respectively, with the corresponding metagenomic database [18]. The numbers of the identified protein were larger than that obtained twice with CelLytic B and CHAPS buffers in our previous study by 1.2–2.2 times [4]. As shown in Table 1, a detailed examination of MS/MS based peptide and protein identifications using Scaffold [19] further indicated that the SDS-, RIPA- and CHAPS-based extraction had higher quantity of unique peptide and MS spectra than the Triton buffer. In addition, 10.6% and 16.5%–21.0% of identified proteins extracted using neutral and ionic detergents, respectively, were membrane proteins. This result is consistent with a previous study, showing that the anionic SDS extraction can produce higher yield of membrane proteins than other ionic detergents [15]. Membrane proteins perform a large variety of functions such as transport, ion channels, enzymatic activity, signal transduction, *etc.*, and usually account for about 20%~30% of the total amount of proteins in the prokaryotic cells [20]. All these observations above suggest that the anionic and zwitterionic detergents can provide better efficiency for recovering the proteins from methane-producing biofilm. 

**Table 1:** Summary of biofilm proteomes obtained using different extraction buffers.

Analysis	Extraction Buffer Containing			
	SDS	Triton X-100	CHAPS	RIPA
Protein recovery (μg/g)	1182.9 ± 196.6	884.8 ± 221.0	1065.4 ± 217.9	1056.1 ± 241.4
Number of identified protein ^1^	591	323	418	473
Proteins ^2^	1206	836	995	1121
Unique peptide number	1955	1061	1407	1395
Unique spectra number	2110	1130	1530	1545
Proteins ^3^	477	317	439	447
Unique peptide number	1345	614	955	988
Unique spectra number	1476	663	1060	1109
Membrane protein (% of total) ^4^	103 (17.5%)	34 (10.6%)	69 (16.5%)	99 (21.0%)

^1^ Using 95% protein probability/95% peptide probability/≥2 peptide by Proteome Discoverer 1.1 (Thermo Scientific, Waltham, MA, USA); ^2^ Using 95% protein probability/95% peptide probability/≥1 peptide by Scaffold _4.0.6.1 (Proteome Software Inc., Portland, OR, USA); ^3^ Using 95% protein probability/95% peptide probability/≥2 peptides by Scaffold_4.0.6.1; ^4^ PSORTb version 3.0.2 [21].

### 2.2. Venn Diagram Analysis

**Figure 2:**
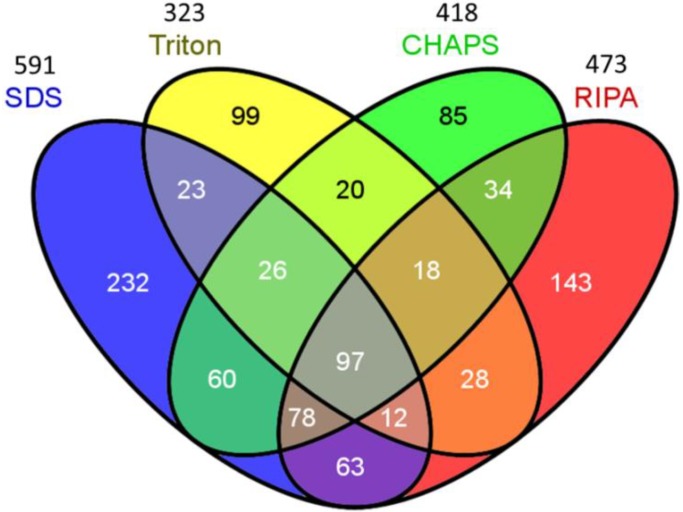
Analysis of the Venn diagram on the identified proteins. (cutoff: protein probability 95%, minimum peptides 2, minimum peptide probability 95%).

Proteins from the four treatments resulted in an identification of total non-redundant proteins up to 1018, suggesting that 31.7%–58.7% of total identified proteins can be recovered with the individual buffer. To further identify unique and common proteins associated with the individual buffers and the combined buffers of two or more, a Venn diagram analysis was performed. Figure 2 displays the results of Venn diagram analysis, illustrating all possible logical relations between a finite collection of sets. The results suggested that totally 97 proteins were common among the proteomes obtained with each buffer, accounting for 9.5% of total identified proteins. Particularly, 8.3%–9.7% of identified proteins were specific to the CHAPS, and Triton buffers, whereas 14.0%–22.8% of identified proteins were unique to SDS and RIPA, respectively. Regarding the proteins unique to pairwise comparisons, the Triton extraction treatment presented the lowest overlap in the identified proteins among the buffers (54.5% with SDS, 55.9% with CHAPS and 59.2% with RIPA), suggesting that the triton buffer extracted the proteins that are dissimilar to other three buffers. This is supported by the results even though a protein identification with one peptide was applied (Figure S1). Combination of RIPA and SDS-based buffers was the most efficient in extracting proteins from biofilm and achieved an identification up to 81.9% of total identified proteins in the LC-MS/MS analysis.

### 2.3. Distribution of Protein Molecular Weight

**Figure 3:**
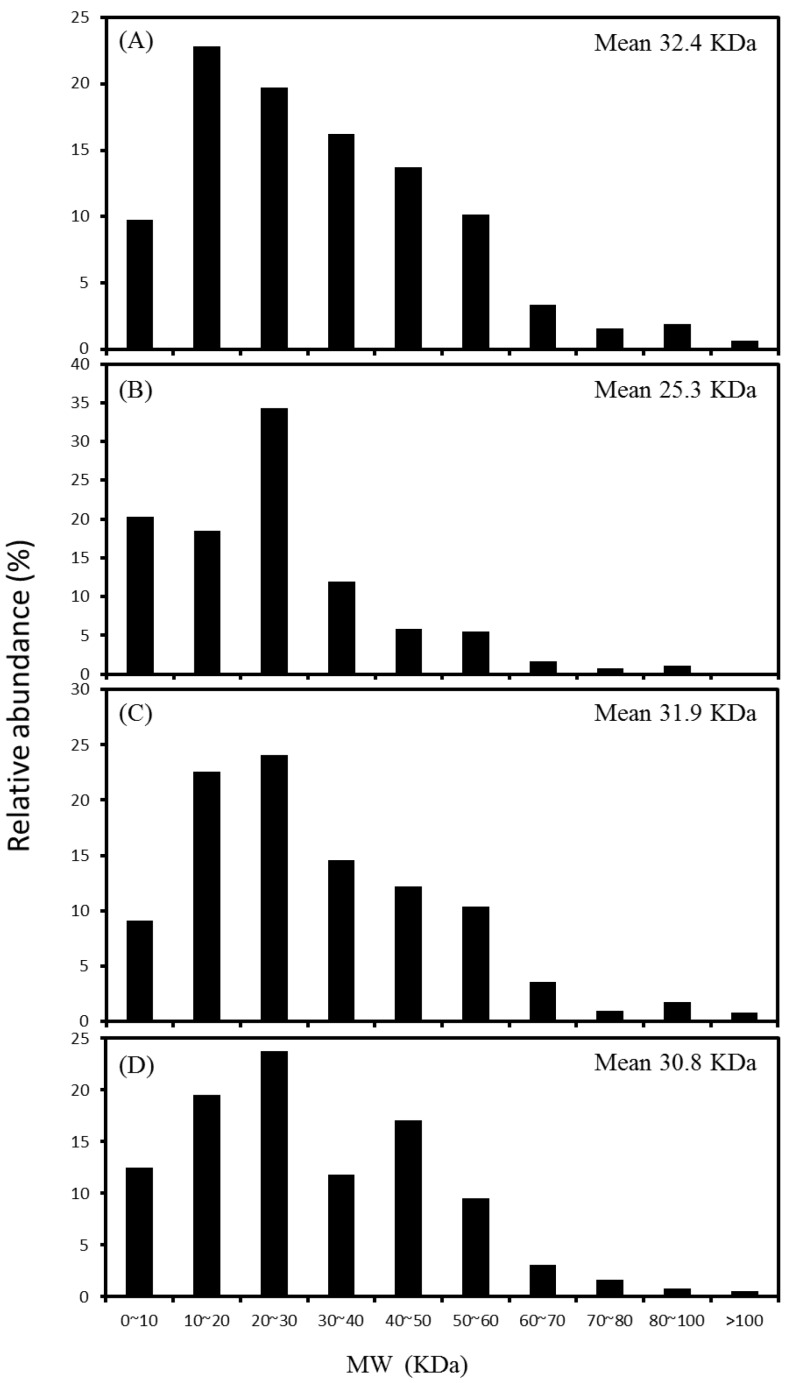
Distribution of molecular weight (*M*_W_) of the identified proteins extracted with SDS (**A**); Triton (**B**); CHAPS (**C**); and RIPA (**D**) buffers.

The extraction method caused a slight selective enrichment on specific sizes of proteins in the obtained proteomes. For proteins with molecular mass in the range of 3–250 kDa, SDS, Triton, CHAPS and RIPA buffers extracted proteins with an average mass of 32.4, 25.3, 31.9 and 30.8 kDa, respectively (Figure 3). More than 76.1% of the identified proteins distributed in the range from 10 to 60 kDa. It was particularly noted that the Triton-based extraction recovered the proteins of 20–30 kDa and below at a higher abundance (73.0%) than other three buffers, suggesting the superiority in the recovery of low molecular weight proteins. In contrast, the proteins of 30–60 kDa accounted for higher percentages (37.1%–40.1%) with the buffers of SDS and RIPA. 

### 2.4. Distribution of Isoelectric Point (pI)

**Figure 4:**
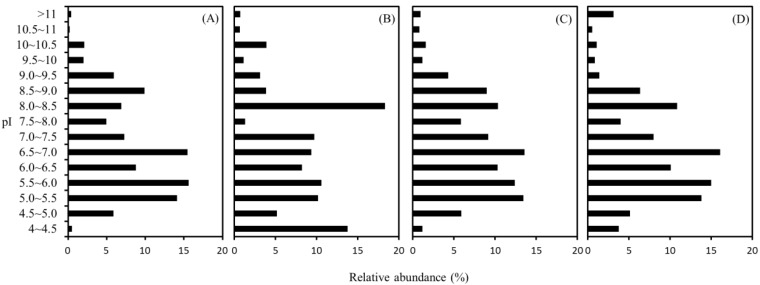
Distribution of isoelectric point (pI) of the identified proteins extracted with SDS (**A**); Triton (**B**); CHAPS (**C**); and RIPA (**D**) buffers.

The pI values that affect the solubility of a protein molecule can be used to evaluate the net charge of the proteins at a given pH. To reveal the effect of the buffer, we thus compared the pI distribution of the identified proteins obtained with the four buffers. As shown in Figure 4, the distribution of pI values of the identified proteins spanned over a broad range from 4.1 to 11.8. It was reported that the proteome of individual microorganisms is usually divided into acidic and basic proteins as a pattern of bimodal distribution, and the fractions of acidic and basic proteins may be correlated with microbial phylogeny and proteome size [22,23,24]. However, in the present work, the distribution of pI displays a trimodal profile with peak frequencies at pH of 5.0–6.0, 6.5–7.0 and 8.0–8.5. It was observed that the clustered pI of methanogens and pyrococcus distributed around pH at 5 to 6 and 8.5 to 9.0 [25,26]. The higher complexity on the pI distribution is likely attributed to the microbial and functional diversity in the methane-producing biofilm analyzed in this study. The highest abundance of proteins occurred on the pH of 6.5–7.0 and 8.0–8.5 for the CHAPS, SDS, and RIPA, and Triton buffers, respectively. In addition, the abundance of the proteins with pI at 4–4.5 is usually low (0.5%–3.8%) using the buffers of SDA, CHAPS, and RIPA, whereas the percentage up to 13.8% was observed for the use of Triton buffer. These observations reveal a high dependence of the pI profile on the characteristics of the extraction buffer. 

### 2.5. Hydrophobicity of Protein

**Figure 5:**
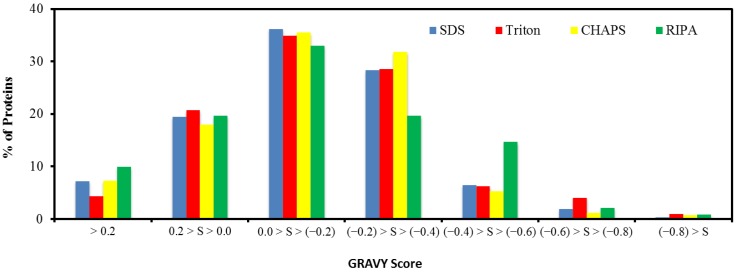
Distribution of the protein grand average of hydropathy (GRAVY) score.

The hydrophobicity that determines the solubility and aggregation of a protein can greatly affect the recovery efficiency in the recovery process [27]. To evaluate the protein hydrophobicity, a grand average of hydropathy (GRAVY) score that is defined by the sum of hydropathy values of all amino acids divided by the protein length was used [28]. Figure 5 shows the results of the GRAVY score distribution, spanning from −0.9 to 1.4. In particular, 70.4%–74.7% of the identified proteins with a GRAVY score within 0 and −1.2 were therefore hydrophilic, whereas the hydrophobic proteins with GRAVY score >0 account for 25.2%–29.6% of the identified proteins. The result suggests that the treatment with the RIPA buffer appeared recovery of the more hydrophobic proteins, and this paralleled the high recovery of membrane proteins using this extraction buffer (Table 1).

### 2.6. Expression of Microbial Functions

**Figure 6:**
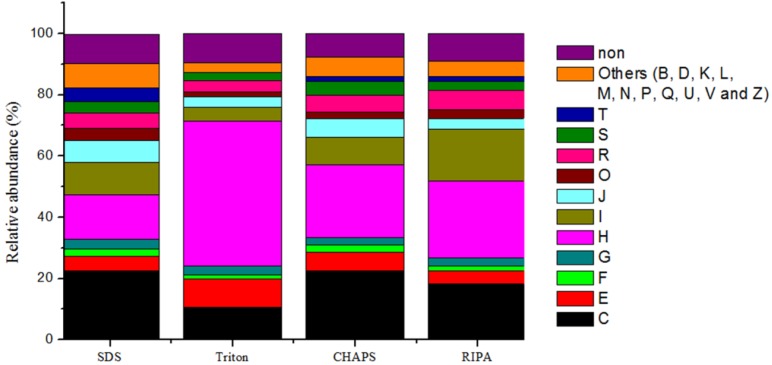
Comparison of the relative abundance of the identified proteins assigned to the functional categories based on cluster of orthologous groups (COG) classification. (Symbols, **C**: Energy production and conversion; **E**: Amino acid transport and metabolism; **F**: Nucleotide transport and metabolism; **G**: Carbohydrate transport and metabolism; **H**: Coenzyme transport and metabolism; **I**: Lipid transport and metabolism; **J**: Translation, ribosomal structure and biogenesis; **K**: Transcription; **L**: Replication, recombination and repair; **M**: Cell wall/membrane/envelope biogenesis; **O**: Posttranslational modification, protein turnover, chaperones; **P**: Inorganic ion transport and metabolism; **R**: General function prediction only; **S**: Function unknown; **T**: Signal transduction mechanisms; **B**: Chromatin structure and dynamics; **D**: Cell cycle control, cell division, chromosome partitioning; **N**: Cell motility; **Q**: Secondary metabolites biosynthesis, transport and catabolism; **U**: Intracellular trafficking, secretion, and vesicular transport; **V**: Defense mechanisms; **Z**: Cytoskeleton, and **non**: unclassified).

To compare the expressed functions of microbial populations, the identified proteins were annotated with cluster of orthologous groups (COG). Based on their assignment, we obtained 498 non-repeated COGs in 22 COG categories. Differences in protein extraction among the extraction buffers reflected differences in microbial functional groups (Figure 6). For examples, higher abundance of proteins (47.3%) was observed in the category H (*i.e.*, coenzyme metabolism) with the Triton buffer, than with the other three buffers (14.6%–25.1%). The relative abundance of proteins assigned to the category C (*i.e.*, energy production and conversion) was also high, with 10.7% of identified proteins of Triton buffer, 18.2% of identified proteins of RIPA buffer and 22.5% of identified proteins of SDS and CHAPS buffers. Only 4.4% of identified proteins obtained with the Triton buffer were assigned to the category I (*i.e.*, lipid metabolism), and lower than the percentage of RIPA buffer by 17.0%. Likewise, using the Triton buffer, only 0.2% of identified proteins were classified to the category T (*i.e.*, signal transduction), whereas up to 4.5% of identified proteins was achieved using the SDS buffer. 

### 2.7. Proteomic-Based Microbial Community Structure

**Figure 7:**
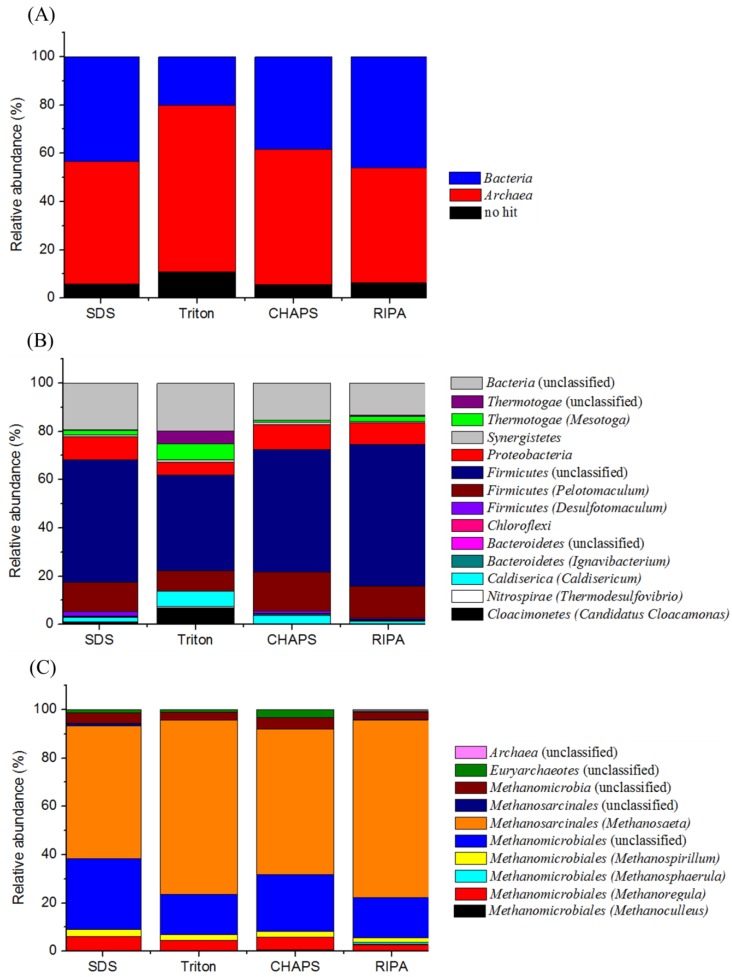
Comparison of the identified proteins that were assigned into the general group (**A**); the domain *Bacteria* (**B**); and the domain *Archaea* (**C**).

To further assess extraction buffers, we compared the relative abundance of spectra at different phylogenetic levels regarding the biofilm consortium that can effectively degrade terephthalate into methane. Figure 7 shows the results obtained with the populations of domains *Bacteria* and *Archaea*. Although it would be difficult to achieve the analysis of replicate samples from the four buffer treatments due to almost running out of the biofilm sampled, in general, the relative abundance of taxa-based identified proteins obtained with the buffers of SDS, CHAPS and RIPA were comparable, but notably different from that treated with the Triton buffer. Proteins related to the archaeal populations accounted for a relative abundance of 47.7%–56.0% of identified proteins, which were generally higher than that (38.4%–46.0%) of the proteins associated with the bacterial populations (Figure 7A). 

For the proteins assigned to the bacterial populations, at least nine phyla were found in the biofilm with each buffer extraction applied (Figure 7B). Among them, the bacterial populations related to the *Firmicutes* were predominant. The relative abundance of proteins, accounting for 64.9%–72.4% of the identified proteins were close with each other among the buffers of SDS, CHAPS and RIPA, but was higher than the percentage (47.9%) associated with the Triton buffer. Within *Firmicutes*, the *Pelotomaculum* spp., capable of degrading terephthalate to acetate and H_2_/CO_2_ syntrophically [4,18] had a relative abundance of proteins at level of 8.3% with the Triton buffer, and in the range 12.4%–16.1% with the SDS, CHAPS, and RIPA buffers (Figure 7B). Despite different methods used, the protein abundance of the *Pelotomaculum* spp. under a terephthalate loading of 0.25 kg·m^−3^·day^−^^1^ in this study was lower than that (87.1% of *Firmicutes*) observed at a terephthalate loading of 2.0 kg·m^−3^·day^−^^1^ [4]. The protein extraction with Triton buffer could allow detecting more abundance of proteins associated with phyla *Thermotogae* (12.1%), and *Caldisericia* (6.4%), as well as candidate division *Cloacimonetes* (6.9%), as compared with the data of anionic and zwitterionic buffers applied. This finding may be related to that the proteins recovered using the Triton buffer exhibited different profiles of molecular weight and pI values from other three buffers applied (Figure 3 and Figure 4). The metaproteomic analysis could identify the functional groups at the genus level, and suggested that the bacterial populations related to the genera *Mesotoga* (56.2% of *Thermotogae*), *Caldisericum* (100% of *Caldisericia*), and candidatus *Cloacamonas* (100% of *Cloacimonetes*) highly dominated in the phyla *Thermotogae*, *Caldisericia* and *Cloacimonetes*, respectively. The metagenomic study predicted that the three bacterial genera were likely involved in the secondary syntrophic interactions in the metabolism of intermediates produced during anaerobic degradations of terephthalate and biomass [18].

Likewise, the differences in the relative abundance of identified proteins belonging to *Archaea* were observed. As shown in Figure 7C, two patterns of phylogeny distribution profiles on the identified archaeal populations were identified, namely the group represented with the buffers of Triton and RIPA, and the other group represented with the buffers of SDS and CHAPS. Based on the shotgun proteomics, the results indicated a dominance of acetotrophic *Methanosaeta* (54.8%–73.5%) and hydrogenotrophic *Methanomicrobiales* (16.8%–29.3%) in the biofilm. These two groups of methanogenic *Archaea* convert the acetate and H_2_/CO_2_ from the syntrophic degradation of terephthalate to the final gaseous products, e.g., methane and CO_2_. Likely, *Methanolinea* was the major member within *Methanomicrobiales* in the biofilm [4]. Another four genera within *Methanomicrobiales*, including *Methanospirillum* (1.8%–2.9%), *Methanosphaerula* (0%–0.9%), *Methanoregula* (2.7%–5.6%), and *Methanoculleus* (0.1%–0.5%) with a relative abundance were detected in this study, but could not be detected using one-dimensional gel-based proteomic analysis [4]. This is likely attributed to the change of microbial community structure accompanied with the varied loading conditions, and/or the better resolution on the microbial community structure associated with detecting higher number of proteins with the current method. Overall, an understanding of those results suggests that the identification of seemingly abundant proteins from a complex biofilm sample may not reflect the true functional dominance, which can in turn bias the interpretation of the shotgun proteomics analysis. 

### 2.8. Clone Library Analysis of the 16S rRNA Gene Sequences

To validate the metaproteomics results, a clone library of bacterial 16S rRNA gene was constructed with the DNA of biofilm. The phylogeny analysis of 16S rRNA gene sequences revealed that the abundant bacterial populations in biofilm were phylogenetically associated with seven bacterial phyla, including *Firmicutes* (30.6% of total clones (127 clones)), *Caldisericia* (9.4% of total clones), *Nitrospira* (8.7% of total clones), *Plactomycetes* (6.4% of total clones), *Proteobacteria* (2.4% of total clones), *Thermotogae* (1.6% of total clones), and *Synergistetes* (0.8% of total clones), as well as two candidate phyla JS1 (13.4% of total clones) and OP8 (7.1% of total clones) (Figure S2). The biofilm consortia sampled at day 1460 shared a slightly different bacterial composition at day 99 [4]. In general, the phylogeny distribution based on the clone library analysis was in accordance with that based on the proteomic analysis. At a finer taxonomic resolution, the clone library results indicated that the bacterial populations related to the genera *Caldisericum* (9.4% of total clones), *Thermodesulfovibrio* (8.7% of total clones), *Pelotomaculum* (5.5% of total clones) and *Mesotoga* (1.6% of total clones) were abundant in biofilm, which was coincided by the results of metaproteomic analysis (Figure 7B). Nevertheless, we observed that certain differences were displayed between the two approaches. The uncultured populations within the candidate phyla JS1 and OP8, and the phylum *Planctomycetes* with high frequencies of 13.4%, 7.1% and 6.4%, respectively were identified in the clone library, but the corresponding proteins were not detected using a metaproteomic method. About 6.9% of identified proteins extracted with a Triton buffer were assigned to Candidatus *Cloacamonas*; however, the relevant 16S rRNA gene sequences were absent from the clone library. The insufficient genomic sequences and low cellular activities of these populations, as well as the PCR amplification bias are likely the causes of the resulting differences. 

## 3. Experimental Section

### 3.1. Bioreactor and Sampling

A laboratory-scale anaerobic biofilm reactor fed with synthetic medium has been operated for more than 1500 days [4]. Terephthalate was used as the sole carbon and energy source, and can be effectively degraded to methane and carbon dioxide by the syntrophic microbial consortia formed on the surface of the ceramic rings (Sera Siporax) inside the reactor. The temperature of the reactor was controlled at 50 °C by circulating the heated water through the water liner. The reactor was operated at a loading of 0.25 kg·m^−3^·day^−^^1^ with hydraulic retention time of two days and an up-flow velocity of 7.6 mL·min^−^^1^ from the operation day 945, and achieved a removal efficiency of terephthalate and total dissolved organic carbon higher than 99% and 96%, respectively. Ten biofilm-covered rings were taken from the reactor at the operation day 1460, frozen immediately in liquid nitrogen, and preserved at −80 °C before analysis.

### 3.2. Protein Extraction

Six rings were completely grounded in a mortar with liquid nitrogen. Then, 1 g of powered sample was mixed with 1 mL of extraction buffer containing the respective detergent in PBS (pH 7.4), 25 µL of 1 M dithiothreitol (DTT) and 10 µL protease inhibitor (Sigma, St Louis, MO, USA) and was subsequently subjected to five cycles of freeze (liquid nitrogen)-thaw (60 °C) treatment. Proteins were precipitated overnight in acetone at −20 °C, centrifuged at 10,000× *g* for 10 min, and resuspended in the rehydration buffer containing the respective detergent in PBS (pH 7.4). In this study, a anionic detergent, sodium dodecyl sulfate (SDS, 1% (*w*/*v*)) (J.T. Baker^®^, Avantor Performance Materials, Coopersburg, PA, USA), a neutral detergent, triton X-100 (Triton, 1% (*w*/*v*)) (Sigma-Aldrich, Castle Hill, Australia), a zwitterionic detergent, 3-[(3-cholamidopropyl)dimethylammonio]-1-propanesulfonate (CHAPS, 1% (*w*/*v*)) (Sigma-Aldrich) was used, respectively. In addition, a RIPA buffer that is an anionic and neutral mixed detergent contains a mixture of Nonidet P-40 (Sigma-Aldrich), 0.1%, sodium deoxycholate (Sigma-Aldrich), 0.5% and SDS, 0.1% in PBS (pH 7.4). The concentration of extracted protein was quantified by 2D-Quant Kit (GE-Amersham Biosciences, St Louis, MO, USA).

### 3.3. In Solution Tryptic Digestion

Prior to a trypsin treatment, samples equivalent to 100 µg protein was pipetted into centrifuge tubes, with each 60 µL, and mixed up with 9.3 µL of 7.5% SDS buffer and 0.7 µL of 1 M DTT. The solution was kept at 95 °C for 5 min to denature the disulfide bonds of proteins. The denatured proteins were then alkylated with 0.5 M iodoacetamide in the dark for 30 min. After alkylation, 52 µL of 50% trichloroacetic acid (TCA) was added into the solution and kept on ice for 15 min to precipitate the proteins. The solution was subsequently centrifuged at 13,800× *g* for 10 min and the pellet was washed with 100 µL of 10% TCA. After centrifuged at 13,800× *g* for 5 min, the pellet was washed three times with deionized water. Finally, the TCA-purified proteins were re-dissolved in 100 μL of 0.1 M ammonium bicarbonate (pH 8.0). The protein molecules were digested enzymatically into short peptide fragments with trypsin (Trypsin Gold, mass spectrometry grade; Promega, Fitchburg, WI, USA) at a protein to trypsin ratio of 50:1 (*w*/*w*) at 37 °C for 16 h [29]. The digest solution was stored in −20 °C, before mass spectrometry analysis. 

### 3.4. On-Line Two Dimensional HPLC-MS/MS Analysis

The separation of proteolytic peptides was performed on a nanoflow-HPLC system (Thermo Finnigan, Surveyor MS Pump Plus, Thermo Scientific). Totally, 80 μg of each sample was loaded, concentrated by a C_18_ trapping column (100 μm i.d. × 5 cm, packed with 5 µm 300 A pore size C_18_ resin), and then subjected to a first dimension separation with a strong cation exchange (SCX) column (5 µm beads, 300 A pore size polysulfoethyl aspartamide strong cation exchange resin; PolyLC, Columbia, MD, USA). The eluent was followed by a second dimension separation with a connected RP-18 column (self-packed New Objective Picofrit 15 cm × 75 µm inner diameter capillary column, containing Jupiter, 5 µm particle size , 300 A pore size C_18_ resin), before entering into a mass spectrometer LTQ Orbitrap XL (Thermo Fisher Scientific, Boston, MA, USA). Buffer A (0.1% formic acid), Buffer B (0.1% formic acid in 80% acetonitrile), and Buffer C (0.1% formic acid in 500 mM ammonia formate) were prepared for the HPLC separation. An elution program was applied by using a gradient to elute the salt from the SCX column for 5 min, followed by an acetonitrile gradient for RP-C_18_ column for 100 min. The details of the gradient elution were: 5 min salt eluted with Buffer C from 0% to 100% (10% interval), and then 90% Buffer C with 10% Buffer B, respectively, in connection with a gradient comprising 0%–10% of Buffer B for 5 min, 10%–40% Buffer B for 85 min, 40%–100% Buffer B for 10 min, and 100% Buffer B for 10 min at a flow rate of 300 nL/min [30].

The peptides were analyzed in the positive ion mode by electrospray ionization (spray voltage = 1.6 kV). One full scan with *m*/*z* 300 to 1800 in the Orbitrap (*R* = 60,000 at *m*/*z* 400) was performed using a rate of 30 ms/scan. The ten most intense peaks were selected for fragmentation with a normalized collision energy value of 35% in the LTQ, and a repeat duration of three minutes was applied to exclude the same *m*/*z* ions from the reselection for fragmentation.

### 3.5. Data Processing and Protein Identification

The obtained mass spectrometry data were analyzed using Proteome Discoverer 1.1 (Thermo Fisher Scientific) with the terephthalate-degrading community metagenome database (Release Date: 3 November 2010) from the integrated microbial genomes and metagenomes (IMG/M) sequence database in Joint Genome Institute [31]. Peptide searching was performed by the SEQUEST algorithm with following parameters: 5 ppm precursor mass measurement accuracy, 0.8 Da product ion mass measurement accuracy, a maximum of two missed tryptic peptides, static modification of Carbamidomethyl (C). The cross correction values (Xcorrs) *versus* charges >1.5 (+1), 2.0 (+2), and 2.5 (+3), only count rank 1 peptide and searching for the top protein were selected for filtering the output data set. Scaffold (version 4.0.6.1, Proteome Software Inc., Portland, OR, USA) was used to validate MS/MS based peptide and protein identifications. To ensure the protein identification, only proteins with at least two peptides identified in the LC-MS/MS analysis were included for analysis.

### 3.6. Protein Annotations and Analysis

The FASTA format of protein sequence was annotated by using stand-alone BLAST program combined with the Conserved Domain Database (CDD) [32], and the Clusters of Orthologous Group (COG) at the National Center for Biotechnology Information (NCBI) [33]. The annotated proteins can be classified to the COG categories within the four groups, including information storage and processing, cellular processes and signaling, metabolism, and poorly characterized. A spectral abundance factor (SAF) where spectral counts were divided by protein length was used to account for the protein abundance [34]. To assess the relative abundance of the proteins, the SAF values were then normalized against the sum of all SAFs for a particular run (removing redundant proteins), for comparison across different buffer treatments. The Venn diagram analysis was performed by a web tool generator [35]*.* Protein hydrophobicity was evaluated based on the grand average of hydropathicity (GRAVY) score [36].

## 4. Conclusions

In the present study, we evaluated the characteristics of the proteins extracted from complex microbial consortia in biofilm with extraction buffers containing different detergents. Results showed that the SDS- and CHAPS-based extraction treatment recovered proteins with high yield and quality, suitable for protein identification using 2D-LC-MS/MS. However, only about up to 54.9% of total proteins could be identified among the individual treatment and each biofilm proteome exhibits the different COG patterns of the proteins, and proteins with different physic-chemical properties. These findings suggest that the extraction buffer can bias the analysis of functional groups, and diversity of protein pattern in biofilms, and the combination of two or more proteins extraction methods, along with proteogenomics analysis is suggested. Furthermore, to gain the better picture of the *in situ* microbial functionality in the biofilm ecosystem, the metaproteomic analysis can be combined with the rRNA-based molecular analysis or other complementary “omic” approach such as metatranscriptomics. Overall, the results of this study provide insight to advance current understanding of the effects of the detergents on the shotgun metaproteomic analysis, and can improve the relevant metaproteomic studies on microbial functions of biofilms in the water and wastewater treatment systems.

## Supplementary Files

Supplementary File 1 Supplementary Information (PDF, 1393 KB)
