# Supplementary material for: Total Protein Extraction for Metaproteomics Analysis of Methane Producing Biofilm: The Effects of Detergents
Source: Int J Mol Sci. 2014 Jun 6;15(6):10169–84. doi: 10.3390/ijms150610169 (PMC4100146; doi:10.3390/ijms150610169)
Supplement: Supplementary File 1 — Supplementary Information (PDF, 1393 KB) [file ijms-15-10169-s001.pdf]

Supplementary Information

Figure S1. Analysis of the Venn diagram on the identifiable proteins (cutoff: protein probability 95%, minimum peptide 1, minimum peptide probability 95%). SDS (sodium dodecyl sulfate), CHAPS (3-[(3-cholamidopropyl)dimethylammonio]-1-propanesulfonate), RIPA (radioimmunoprecipitation assay buffer).

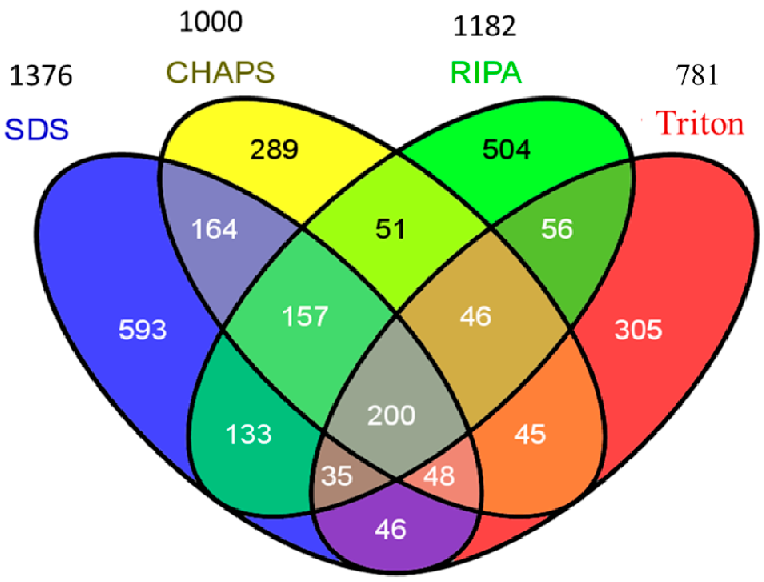

Figure S2. Phylogenetic tree of the 16S rRNA gene sequences of bacterial populations in the anaerobic terephthalate-degrading biofilm under 50 °C conditions. Total community DNA subjected to the microbial community anlaysis was obtained from biofilms sampled at Day 1460. The tree was reconstructed based on the neighbor-joining method with 1000-time bootstrapping and rooted with *Methanospirillum hungatei* (CP000524). Bootstrap values that are greater than 60% are indicated at branch nodes using a solid circle. The scale bar corresponds to 10 nucleotide substitutions per 100 nucleotide positions. The phylotypes obtained in this study and in our previous study [1] were highlighted in blue and red, respectively. The frequencies of phylotypes in the clone library were indicated in parentheses. The inference of taxonomic phyla for the phylotypes was showed on the right of the tree.

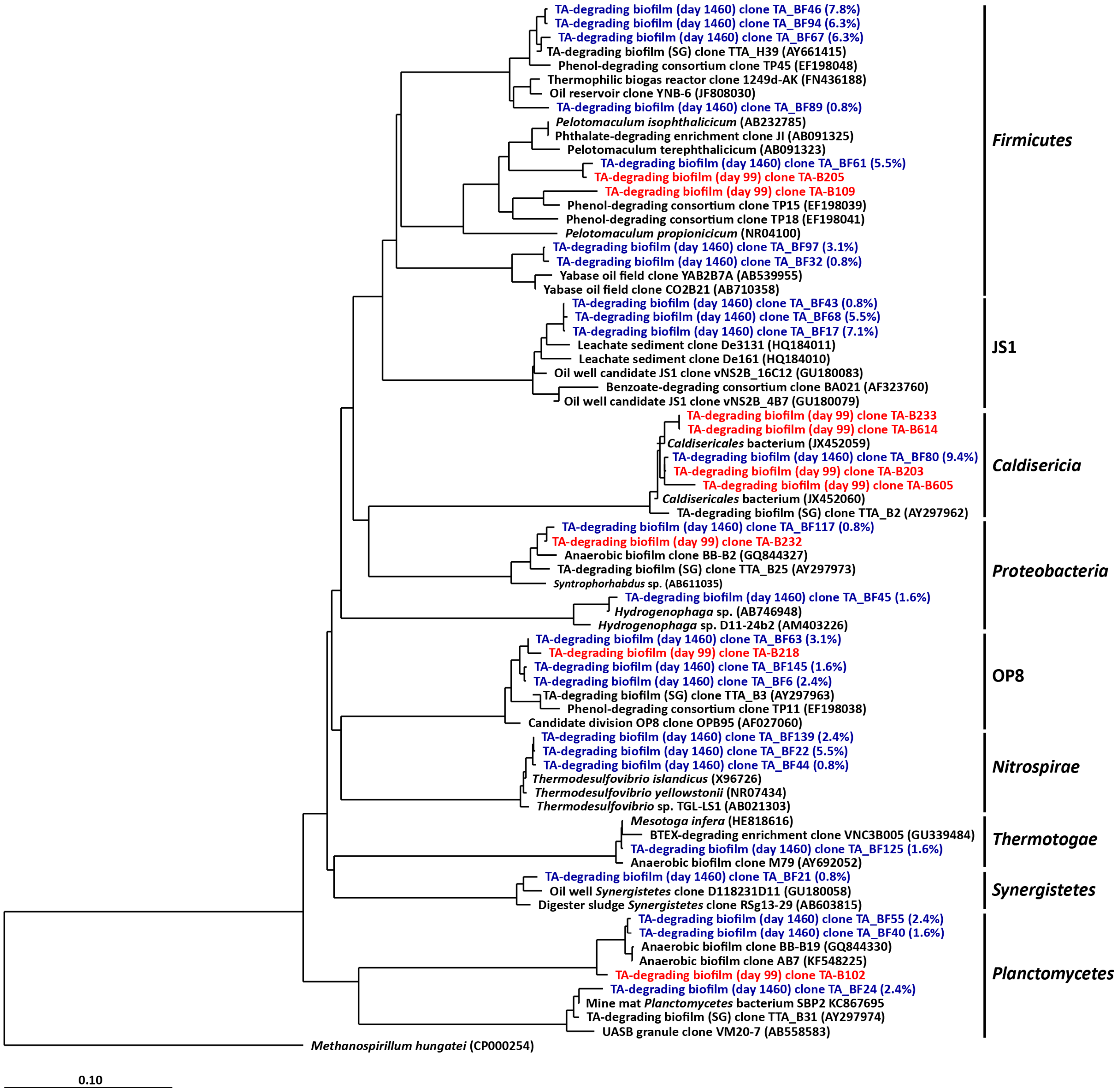

Biofilm DNA preparation and bacterial composition analysis

For preparation of genomic DNA from biofilm samples, microbial cells were subjected to enzymatic and chemical treatments, and their community DNAs were purified and precipitated using phenol-chloroform-isoamyl alcohol and ethanol precipitation procedures [2]. Prior to analysis of microbial composition, the bacterial 16S rRNA gene fragments were obtained by PCR amplification with the commonly used primer set (27F/1492R), and cloned using a TOPO TA cloning kit (Invitrogen, Carlsbad, CA, USA). The clones were screened using the restriction fragment length polymorphism (RFLP) method, and those sharing a RFLP profile were grouped into one operational taxonomic unit (OTU). The sequences of each OTU representative were analyzed in accordance with our previous study [3]. The nucleotide sequences of the detected OTUs were assessed for chimeric artifacts using Pintail software [4], and then compared with the sequences of relatives in the GenBank using a BLAST search. The phylogenetic tree and bootstrap resampling analysis for 1000 replicates were constructed by applying the neighbor-joining method provided in ARB program [5].

References

1. Wu, J.H.; Chuang, H.P.; Hsu, M.H.; Chen W.Y. Multiplexed relative abundance analysis of methanogens in anaerobic digestion systems using a hierarchical oligonucleotide primer extension approach. *Appl. Environ. Microbiol.* **2013**, *79*, doi:10.1128/AEM.02450-13.
2. Wu, J.H.; Liu, W.T.; Tseng, I.C.; Cheng, S.S. Characterization of microbial consortia in a terephthalate-degrading anaerobic granular sludge system. *Microbiology* **2001**, *147*, 373–382.
3. Chen, C.L.; Wu, J.H.; Tseng, I.C.; Liang, T.M.; Liu, W.T. Characterization of active microbes in a full-scale anaerobic fluidized bed reactor treating phenolic wastewater. *Microbes Environ.* **2009**, *24*, 144–153.
4. Ashelford, K.E.; Chuzhanova, N.A.; Fry, J.C.; Jones, A.J.; Weightman, A.J. At least 1 in 20 16S rRNA sequence records currently held in public repositories is estimated to contain substantial anomalies. *Appl. Environ. Microbiol*. **2005**, *71*, 7724–7736.

5 Ludwig, W.; Strunk, O.; Westram, R.; Richter, L.; Meier, H.; Yadhukumar; Buchner, A.; Lai, T.; Steppi, S.; Jobb, G.; *et al.* ARB: A software environment for sequence data. *Nucleic Acids Res.* **2004**, *32*, 1363–1371.

© 2014 by the authors; licensee MDPI, Basel, Switzerland. This article is an open access article distributed under the terms and conditions of the Creative Commons Attribution license (http://creativecommons.org/licenses/by/3.0/).
